# DC-SIGN and Toll-like receptor 4 mediate oxidized low-density lipoprotein-induced inflammatory responses in macrophages

**DOI:** 10.1038/s41598-017-03740-7

**Published:** 2017-06-12

**Authors:** Ke Yang, Xinhe Liu, Yan Liu, Xinqiong Wang, Lijuan Cao, Xiaojie Zhang, Chundi Xu, Weifeng Shen, Tong Zhou

**Affiliations:** 1 Department of Pediatrics, Ruijin Hospital, Shanghai Jiaotong University School of Medicine, Shanghai, 200025 China; 2 Institute of Cardiovascular Disease, Ruijin hospital, Jiaotong University School of Medicine, Shanghai, 200025 China; 3 Department of Cardiology, Shanghai Ninth People’s Hospital, Shanghai Jiaotong University School of Medicine, Shanghai, 200025 China

## Abstract

The regulation of inflammatory responses by innate immune receptors is recognized as a crucial step in the development of atherosclerosis, although the precise molecular mechanisms remain to be elucidated. This study focused on illustrating the roles of dendritic cell-specific intercellular adhesion molecule-3-grabbing non-integrin (DC-SIGN)- and Toll-like receptor 4 (TLR4)-regulated inflammatory responses in macrophages. We found that DC-SIGN expression levels were increased in macrophages of atherosclerotic plaques. Oxidized low-density lipoprotein (oxLDL) significantly enhanced DC-SIGN protein expression levels after a short-term exposure. Knockdown of DC-SIGN decreased expression and secretion of interleukin 1-β (IL1-β), monocyte chemo-attractant protein 1 (MCP-1), tumor necrosis factor-α (TNFα) and matrix metalloproteinase-9 (MMP-9). Immunofluorescence studies demonstrated that DC-SIGN and TLR4 co-localized in regions of the plaques. Moreover, DC-SIGN was co-expressed with TLR4 on the plasma membrane after oxLDL stimulation. The presence of an endogenous interaction and the results of the *in vitro* pull-down assays revealed that DC-SIGN binds directly with TLR4. We also present evidence that DC-SIGN mediates TLR4-regulated NFκB activation but not activation of p38 and JNK. Our results suggest an essential role of DC-SIGN/TLR4 signaling in macrophages in the pathogenesis of atherosclerosis.

## Introduction

Atherosclerosis is a disease exhibiting chronic inflammatory and fibroproliferative responses^1^, which are accelerated by high levels of serum lipids and correlated with risks of cardiovascular disease^2^. In the vascular walls, oxidation of low-density lipoprotein is a main pathological factor contributing to atherosclerosis^3^. Oxidized low-density lipoprotein (oxLDL) leads to the initiation and progression of atherosclerosis^4–6^. Previous studies have found that oxLDL promotes the inflammatory response of macrophages and endothelial dysfunction^7, 8^. Innate immune cells, such as monocyte-macrophages and dendritic cells, have been found in plaque areas, and these cells enhance the progression of atherosclerosis by activating the innate immune system^9–11^. However, the process of innate immune activation requires phagocytes expressing cell surface receptors to bind and respond to the stimuli.

Pattern recognition receptors (PRRs) present on the surface of innate immune cells are a class of receptors for pathogen-associated molecular patterns (PAMPs) and include Toll-like receptors and C-type lectin receptors^12–14^. Dendritic cell-specific intercellular adhesion molecule-3-grabbing non-integrin (DC-SIGN) is a type-II transmembrane lectin receptor^15^. DC-SIGN is abundantly expressed on immature DCs and monocyte-macrophages. Previous studies have demonstrated that DC-SIGN participates in the pathology of atherosclerosis. Plaques from human coronary and carotid arteries and the aorta contain DC-SIGN-immunoreactive cells, which show a co-expression of DC-SIGN and macrophage/DC lineage markers^16^. Elevated plasma homocysteine, an independent risk factor for atherosclerosis, promotes the expression of DC-SIGN on DCs in a concentration-dependent manner^17^. The capacity for the tissue Renin-angiotensin system (RAS) to induce the recruitment of T cells and increase their ability to bind to DCs via DC-SIGN may be one aspect of the pathogenesis of atherosclerosis^18^. Toll-like receptor 4 (TLR4) is expressed on macrophages and DCs^19, 20^ and regulates the inflammatory response in atherosclerosis^21, 22^. There are multiple mechanisms involved in the oxLDL-induced inflammatory response; however, a recent study found that TLR4 is expressed in the lipid-rich region of plaques^23^. In TLR4-deficient mice, the extent of atherosclerosis is significantly decreased, suggesting that TLR4, as a receptor of oxLDL, is involved in the inflammatory response and pathophysiology of atherosclerosis^24, 25^. NF-kB activation also participates in the DC-mediated immune responses^26^. Moreover, TLR increases p65 activation via both MyD88- and TRIF-dependent pathways, which induce the transcription of inflammatory cytokines and chemokines^27^. Yeasts and viruses induce the Raf-1 pathway through DC-SIGN to modulate TLR responses^28^. Strikingly, DC-SIGN signaling controls p65 activity via the phosphorylation of p65 at serine 276 (Ser276), which is completely dependent on Raf-1-activation^29^.

DC-SIGN is involved in Toll-like receptor (TLR)-induced signaling, but the mechanism is not clear. In this study, we show that the binding of DC-SIGN to TLR4 mediates TLR4-induced NF-kB activation in the activation of macrophages. Using immunohistochemistry, we show that DC-SIGN and TLR4 are co-localized on macrophages in human atherosclerotic plaques. OxLDL induces the binding of DC-SIGN to TLR4, as revealed by pull-down assays and immunoprecipitation. We further demonstrate that DC-SIGN regulates the downstream TLR4 pathway under oxLDL and LPS stimulation. These results provide a novel pathway that advances the understanding of the inflammatory response of macrophages in atherosclerosis.

## Materials and Methods

### Reagents and antibodies

OxLDL (oxLDL, Serotec, UK) was purchased and used to stimulate macrophages. Small interfering RNA (siRNA) was used for the knockdown of DC-SIGN (SMARTpool TM, Dharmacon, USA). Interleukin 1-β (IL1-β), monocyte chemo-attractant protein 1 (MCP-1), tumor necrosis factor-α (TNFα) and matrix metalloproteinase-9 (MMP-9) ELISA kits were obtained from R&D Systems (R&D Systems, USA). Immunohistochemical antibodies used to detect DC-SIGN, TLR4, CD68, FLAG tag and His tag were obtained from Abcam (MA, USA). Fetal bovine serum (FBS), RPMI 1640 and Dulbecco’s MEM (DMEM) culture media, and streptomycin and penicillin were purchased from Gibco BRL (Carlsbad, USA). Primary antibodies for DC-SIGN, TLR4, α-tubulin, anti-FLAG, anti-His (Abcam, USA), p65 (t-p65), phosphorylated-p65 (p-p65), IKKε (t- IKKε), phosphorylated-IKKε (p- IKKε), p38 MAPK (t-p38), phosphorylated-p38 MAPK (p-p38), c-Jun N-terminal kinase (t-JNK), and phosphorylated-JNK (p-JNK) were purchased from Cell Signaling Technology (MA, USA). HRP-conjugated antibodies and Alexa 594-, 647- and 488-conjugated antibodies (Cell Signaling Technology) were used as secondary antibodies. pcDNA3.1 (−)/myc-HisA (Invitrogen, USA) and C-terminal pFLAG-CMV-5.1 (Sigma-Aldrich, USA) were used to overexpress TLR4 and DC-SIGN, respectively. Anti-FLAG M2 and Anti-His agarose beads were purchased from Sigma-Aldrich. Normal mouse and rabbit IgGs (eBioscience, USA) were used as negative controls.

### Clinical samples

Internal thoracic arteries (n = 3) without any atherosclerosis were collected from the patient as excess graft material in coronary artery bypass grafting (CABG), and the internal thoracic arteries were used as normal artery controls. In addition, femoral arteries with angiographic atherosclerotic plaques were obtained from another 3 patients who underwent leg amputation (for the clinical information, see Table 1).

**Table 1:** Baseline characteristics and biochemical measurements of atherosclerosis patients.

	Patients (n = 3)
Male gender (n, %)	3 (100)
Age (yrs)	59 ± 1
Cigarette smoking (n, %)	2 (66.7)
Hypertension (n, %)	2 (66.7)
Systolic BP (mmHg)	146 ± 21
Diastolic BP (mmHg)	87 ± 15
Type 2 diabetes (n, %)	3 (100)
BMI (kg/m2)	29.0 ± 0.6
Total cholesterol (mmol/L)	4.76 ± 1.43
HDL-cholesterol (mmol/L)	1.05 ± 0.33
LDL-cholesterol (mmol/L)	3.62 ± 0.77
Triglycerides (mmol/L)	1.09 ± 0.60
Fasting glucose (mmol/L)	4.83 ± 2.06
HbA1c (%)	6.63 ± 1.18
Blood urea nitrogen (mmol/L)	5.13 ± 0.80
Creatinine (umol/L)	81.33 ± 17.50

This study was approved by the Institutional Review Board of RuiJin Hospital, Shanghai Jiaotong University School of Medicine. All patients provided written informed consent, and clinical investigation was conducted according to the principle of the Declaration of Helsinki.

### Cell culture

The whole blood was collected from the healthy donors, and then the monocytes were isolated by Ficoll-Paque separation (GE Healthcare). The collected monocytes were differentiated in the presence of 100 ng/ml M-CSF (PeproTech, London, U.K.) for 3 days. Non-adherent cells were washed off, and the remaining adherent cells (monocytes derived macrophages) were maintained in RPMI 1640 medium supplemented with 20% heat-inactivated fetal calf serum, 1% penicillin/streptomycin, and 2% l-glutamine. To overexpress recombinant human DC-SIGN (with FLAG) and TLR4 (with His) proteins, HEK293 cells (ATCC, U.S.A.) were seeded onto 60-mm dishes at a density of 5.0 × 10^5^ cells and cultured in DMEM medium containing 10% FBS.

### Immunohistochemistry

The human femoral arteries (n = 3) and internal thoracic arteries (n = 3) underwent histological and immunochemical analyses. After fixation in 4% paraformaldehyde overnight, the samples were cut into serial cryosections (5-μm thick). Sections were analyzed by hematoxylin and eosin (H&E) staining or immunofluorescence. Samples were incubated with anti-TLR4 (1:50), anti-DC-SIGN (1:50) or anti-CD68 (1:50) antibodies at 4 °C for 12 hours and then incubated with Alexa 594-, 647- and 488-conjugated secondary antibodies (1:1000). All sections were observed and photographed microscopically (Zeiss Microsystems).

The macrophages were treated with oxLDL to localize DC-SIGN and TLR4. The macrophages (1 × 10^4^/well) were seeded onto an 8-well EZ-SLIDE (Millipore). After stimulation with oxLDL (50 μg/ml) for 6 hours, the macrophages were washed with PBS twice and fixed in 4% paraformaldehyde. Cells were immunostained with either the anti-DC-SIGN (1:50) or TLR4 antibody (1:50) antibody at 4 °C for 12 hours and incubated with Alexa 594-conjugated secondary antibody (1:1000) or Alexa 488-conjugated secondary antibody (1:1000). All cell samples were observed and photographed microscopically (Zeiss Microsystems).

The imaging was performed with ZEISS LSM 800, filters was 410–473, objective is EC Plan-Neofluar 40x/1.30 Oil DIC M27, imaging mode is sequential. The primary antibodies of anti-TLR4 is mouse, anti-DC-SIGN is rabbit and anti-CD68 is goat. The secondary antibodies of Alexa-488 conjunction is Donkey anti-mouse IgG, Alexa-594 conjunction is Donkey anti-Rabbit IgG and Alexa-647 conjunction is Donkey anti-Goat IgG. The colocalization was quantified with Axiovision software (Zeiss MicroImaging, Oberkochen, Germany). Images of entire sections were captured, typically 25 mm^2^ in size, at a resolution of 1 pixel/μm^2^. After acquired images, all pixels having the same positions in both images are considered a pair. Of every pair of pixels (P1, P2) from the two source images, the intensity level of pixel P1 is interpreted as X coordinate, and that of pixel P2 as Y coordinate of the scatter diagram. Selecting all of the pixels that were 2.5 SD above background levels, and the TLR4 and DC-SIGN layers were thresholded, positive regions were identified. Results are representative of 3 independent experiments and showed as integrated optical density (IOD)/area. (n = 3, Mean ± SD, compared with control P < 0.05 as significantly change).

The HEK293 cells (1 × 10^4^/well) were seeded onto an 8-well EZ-SLIDE (Millipore) and then transfected with pcDNA3.1 (−)/myc-HisA, pcDNA3.1 (−)/myc-HisA-TLR4, pFLAG-CMV-5.1 or pFLAG-CMV-5.1-DC-SIGN for 72 hours. The cells were washed with PBS twice and fixed in 4% paraformaldehyde. The pcDNA3.1 (−)/myc-HisA- and pcDNA3.1 (−)/myc-HisA-TLR4-transfected cells were immunostained with anti-TLR4 (1:50) and anti-His (1:100) antibodies at 4 °C for 12 hours and incubated with Alexa 594- and 488-conjugated secondary antibodies (1:1000). The pFLAG-CMV-5.1- and pFLAG-CMV-5.1-DC-SIGN-transfected cells were incubated with the anti-DC-SIGN (1:50) and anti-FLAG (1:100) antibodies at 4 °C for 12 hours and then incubated with Alexa 594- and 488-conjugated secondary antibodies (1:1000). All cell samples were observed and photographed microscopically (Zeiss Microsystems).

### Oligonucleotide and plasmid transfection

Negative control (NC) or DC-SIGN-specific siRNA (100 nM/10^5^ cells) was transfected into macrophages using Lipofectamine reagent (Invitrogen).

The full-length DC-SIGN (1212 bp) and TLR4 (2520 bp) were cloned into pFLAG-CMV-5.1 and pcDNA3.1 (−)/myc-HisA plasmids, respectively, to overexpress FLAG-DC-SIGN and His-TLR4. Transient transfection of HEK293 cells with either pFLAG-CMV-5.1-DC-SIGN or pcDNA3.1 (−)/myc-HisA-TLR4 plasmids (25 μg/10^5^ cells) was performed using Lipofectamine reagent (Invitrogen).

### Immunoblotting and immunoprecipitation

The ProteoJET Mammalian Cell Lysis Reagent (Fermentas, MD, USA) was used to lyse cells to extract cytoplasmic proteins. Equal amounts of lysate (50 μg) were loaded and separated by 12% SDS/PAGE and blotted onto a poly(vinylidene difluoride) membrane. The membrane was blocked with 5% BSA (bovine serum albumin) and then probed with antibodies against DC-SIGN (1:500), TLR4 (1:500), α-tubulin (1:2000), total p65 (1:1000), phosphorylated p65 (1:1000), total IKKε (1:1000), phosphorylated IKKε (1:1000), total p38 (1:1000), phosphorylated p38 (1:1000), total JNK (1:1000), phosphorylated JNK (1:1000), anti-FLAG (1:2000) and anti-His (1:2000) overnight at 4 °C. Then, the membrane was incubated with horseradish peroxidase-conjugated secondary antibodies (1:5000) for 1 hour at room temperature. Blots were developed with an ECL detection system (Millipore, MA, USA).

The interaction between DC-SIGN and TLR4 was detected using a Pierce^®^ Crosslink Immunoprecipitation Kit following the manufacturer’s protocol. Immunoblots were then performed using antibodies diluted in 1% BSA TBST. Antibodies against TLR4 (1:500 dilution) were used. Horseradish peroxidase-conjugated secondary antibodies (Cell Signaling Technologies) were used to visualize the immunoblots.

Each image was captured, and the intensity of each band was analyzed with Quantity One software (Bio-Rad).

### *In vitro* pull-down assay

The HEK293 cells were transfected with pcDNA3.1 (−)/myc-HisA-TLR4, pFLAG-CMV-5.1 or pFLAG-CMV-5.1-DC-SIGN for 72 hours. Then, the total cell lysate (10 μg) of pFLAG-CMV-5.1- or pFLAG-CMV-5.1-DC-SIGN-transfected cells was adsorbed onto a 50-μl bed of anti-FLAG M2 beads. After six washes by centrifugation, the total cell lysate (10 μg) content of His-TLR4 in the equilibration buffer (140 mM NaCl, 10 mM Na_2_HPO_4_, 1.8 mM KH_2_PO_4_, pH 7.5) was incubated with a mixture of protease inhibitors (Roche) overnight at 4 °C. Finally, the bound proteins were washed six times by centrifugation and eluted from the beads with the FLAG peptide (100 mg/ml, Sigma, Cat# F3290) in TBS buffer (50 mM Tris-HCl, 150 mM NaCl, pH 7.4). Eluates were analyzed by SDS-PAGE followed by immunoblotting with anti-His (1:2000), anti-FLAG (1:2000) and anti-TLR4 (1:500).

### Real-time PCR

Quantification of mRNA levels was conducted using Power SYBR Green PCR Master Mix (Applied Biosystems, CA, USA) and a StepOne system (Applied Biosystems). For DC-SIGN, IL1-β, TNFα, MCP-1 and MMP-9 mRNA, each reaction consisted of two stages. The first stage consisted of 30 seconds of denaturation at 95 °C. The second stage consisted of 40 cycles of 10 seconds of denaturation at 95 °C and 31 seconds of extension at 65 °C. The data were analyzed by StepOne software v2.1 (Applied Biosystems), and expression levels were normalized to β-actin levels. The sequences of oligonucleotides used for real-time PCR are listed in Table 2. Primer validation was tested by analyzing the melting curve for the specificity and the amplification curve for the efficiency of the primer (Supplementary Fig. 1).

**Table 2:** The primer has been used for Real-time PCR.

Gene	Forward primer	Reverse primer	products size (bp)
DC-SIGN	5′-TTGGCTGGGCTCCTTGT-3′	5′-CGTCTTGCCTGGATTGTT-3′	70
IL-1β	5′-ACAGTGGCAATGAGGATG-3′	5′-TGTAGTGGTGGTCGGAGA-3′	129
TNF-a	5′-CGAGTGACAAGCCTGTAGCC-3′	5′-TGAAGAGGACCTGGGAGTAGAT-3′	171
MCP-1	5′-TGTGCCTGCTGCTCATAG-3′	5′-TCTTTGGGACACTTGCTG-3′	163
MMP9	5′-CCCCGATGCTGATACTGA-3′	5′-CTGTCCGCCAAATAAACC-3′	152
α-tublin	5′-CGTGGACATCCGCAAAG-3′	5′-TGGAAGGTGGACAGCGA-3′	201

### EMSA

The EMSA was used to detect the activation of p65 binding to DNA. The double-stranded of p65 oligonucleotide probe was radiolabeled with [c-32P] ATP using T4 polynucleotide kinase (Invitrogen). The nuclear extracts of the cells were prepared with NE-PER™ Nuclear and Cytoplasmic Extraction Reagents (ThermoFisher, USA). The nuclear extracts (20 μg) were incubated with the 32P-labeled oligonucleotide in reaction buffer containing 10 mM HEPES (pH 7.9), 70 mM NaCl, 1 mM DTT, 12.5% glycerol, 1 mM EDTA and 2 mg poly(dI-dC) for 20 min at room temperature. The DNA-protein complex was resolved from free oligonucleotides by electrophoresis on 6.6% native polyacrylamide gels.

### Statistical analysis

The data are presented as the mean ± SD. *In vitro* cell experiments were repeated a minimum of six times. Differences between groups were tested using a one-way ANOVA with Dunnett’s C post hoc test. A two-sided probability level of P < 0.05 was used to determine statistical significance. All analyses were performed with SPSS for Windows 13.0.

## Results

### Macrophages in atherosclerotic plaques show high levels of DC-SIGN expression

**Figure 1:**
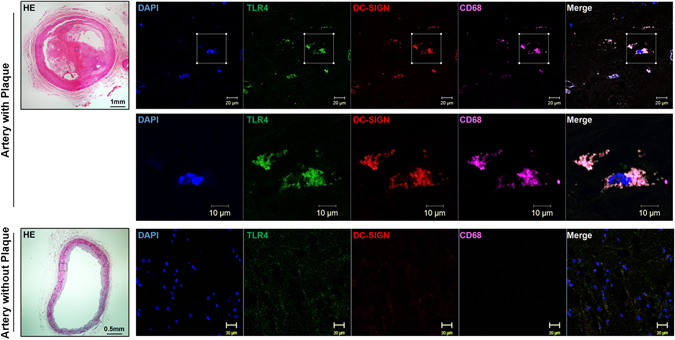
DC-SIGN is expressed in the macrophages of plaques and the macrophages of atherosclerotic patients. Human femoral arteries from patients with angiographic atherosclerotic plaques and internal thoracic arteries without plaques were assessed by histological and immunochemical analysis. As a continuity study, the origin of tissues is same as our previous study^22^. Sections were stained with hematoxylin and eosin or immunofluorescence stains for DC-SIGN, TLR4 and CD68.

The femoral arteries from patients with femoral artery stenosis and internal thoracic arteries (as a control) were stained with hematoxylin and eosin to investigate the pathology of atherosclerosis. The human femoral artery plaques showed a significant amount of macrophage rupture and lipid deposition near the lipid core of the plaque (Fig. 1). Baseline characteristics of atherosclerosis patients are presented in Table 1. The expression and cellular localization of DC-SIGN and TLR4 in atherosclerotic lesions were investigated by immunofluorescence analysis. Macrophages (CD68-positive cells) were enriched around the lipid core of atheroma, which also expressed high levels of DC-SIGN and TLR4 (upper of Fig. 1). In the control arteries, there were no DC-SIGN and TLR4 expressing macrophages (CD68-positive cells) (bottom row of Fig. 1). The control pictures for autofluorescence and other immunofluorescence of control and atherosclerosis arteries are shown in Supplementary Fig. 2.

### OxLDL promotes DC-SIGN expression

**Figure 2:**
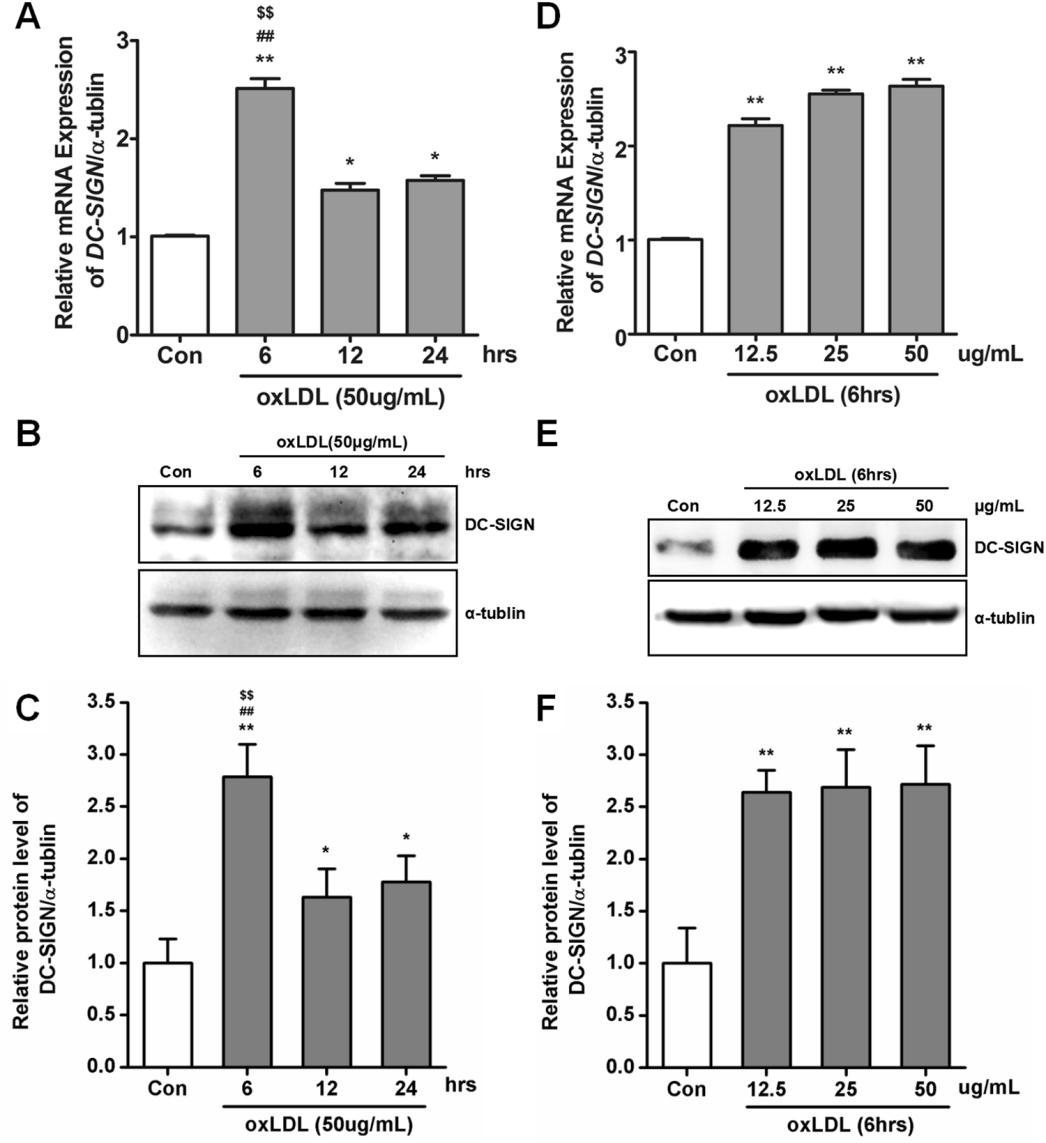
oxLDL-induced DC-SIGN expression. Human primary macrophages were incubated with oxLDL for increasing time intervals (0, 6, 12 and 24 hours with 50 μg/ml) or increasing doses (0, 12.5, 25 and 50 μg/ml for 6 hours). The expression level of DC-SIGN was detected by real-time PCR (**A** and **D**) and western blot analysis (**B** and **E**) and quantified by densitometry as relative units (DC-SIGN/α-tubulin) in 3 independent experiments (**C** and **F**). The data are expressed as the mean ± SD from 3 independent tests. *P < 0.05, **P < 0.01 compared with human primary macrophages not treated with oxLDL, ^##^ *P* < 0.01 compared with human primary macrophages not treated with oxLDL within 12 hours, ^$$^ *P* < 0.01 compared with human primary macrophages not treated with oxLDL within 24 hours.

According to the immunofluorescence results in atherosclerotic patients, we examined whether DC-SIGN is involved in oxLDL-induced macrophage activation. Primary macrophages were incubated with oxLDL (for 6, 12 or 24 hours and at concentrations of 12.5, 25 or 50 μg/ml) to measure its effects on DC-SIGN expression (Fig. 2A–F). After 6 hours of incubation with 50 μg/ml of oxLDL, further treatment did not significantly increase mRNA and protein expression of DC-SIGN (6 hours: 2.79-fold, 12 hours: 1.63-fold; and 24 hours: 1.77-fold compared to that of control, *P* < 0.05, Fig. 2A–C). Different doses of oxLDL increased DC-SIGN mRNA and protein expression to similar levels (12.5 μg/ml: 2.64-fold, 25 μg/ml: 2.69-fold; and 50 μg/ml: 2.72-fold compared to that of control, *P* < 0.05, Fig. 2D–F). In conclusion, these data show that in primary macrophages, the DC-SIGN level was rapidly regulated by oxLDL stimulation.

### Knockdown of DC-SIGN expression inhibits oxLDL-induced inflammatory cytokine production

**Figure 3:**
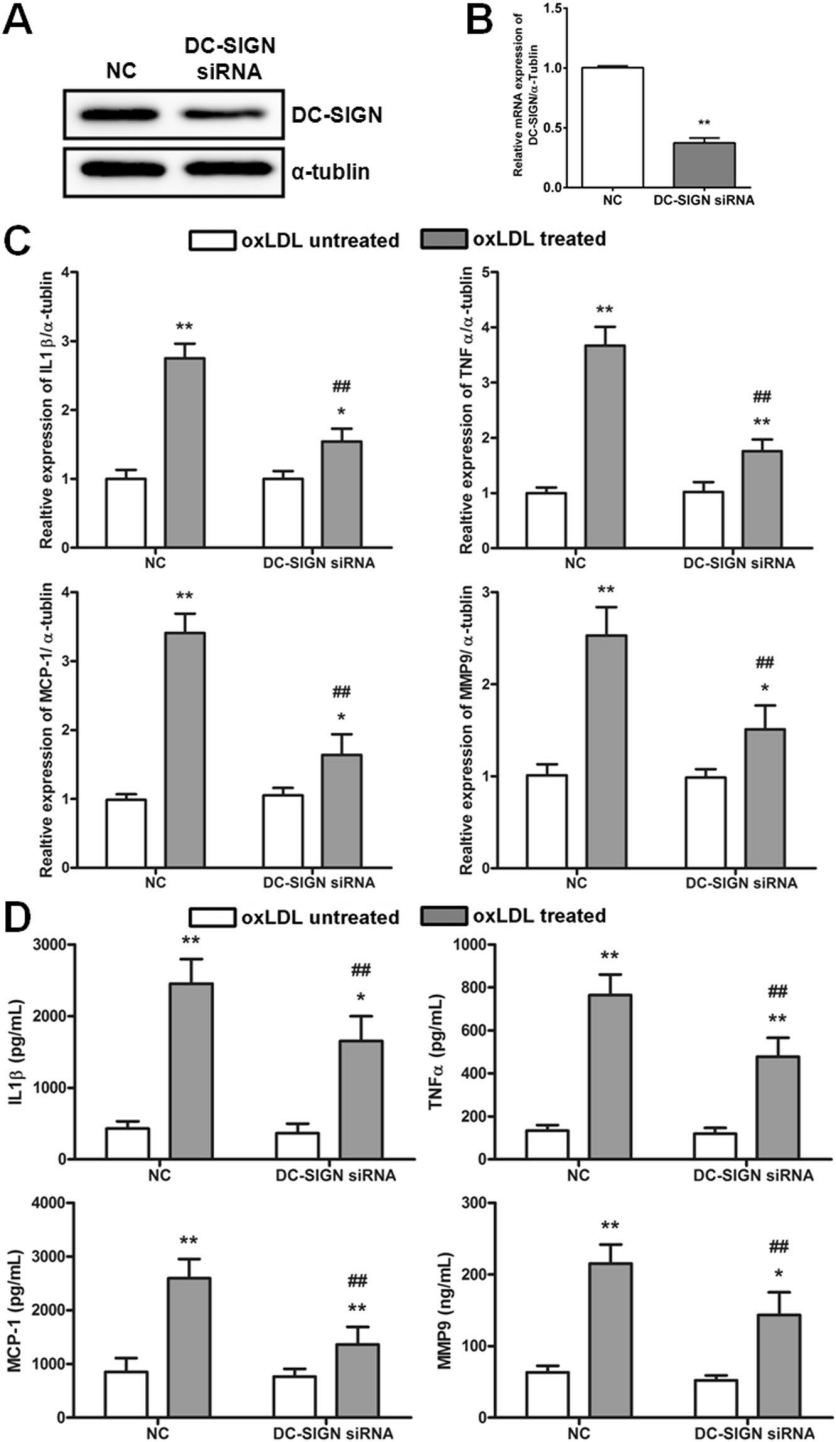
DC-SIGN-regulated inflammatory cytokine expression. DC-SIGN or a negative control (NC) siRNA was transfected into macrophages treated or not treated with oxLDL (50 μg/ml) for 6 hours. (**A**) The knockdown efficiency of DC-SIGN siRNA was detected by western blot analysis. (**B**) The efficiency of DC-SIGN knockdown was detected by real-time PCR. The data are expressed as the mean ± SD from 3 independent tests. **P < 0.01 compared with the macrophages transfected with negative control siRNA (NC). (**C**) Real-time PCR detected the expression of IL-1β, TNF-α, MCP-1 and MMP-9. (**D**) ELISA measured the secretion of IL-1β, TNF-α, MCP-1 and MMP-9. The data are expressed as the mean ± SD from 3 independent tests. *P < 0.05, **P < 0.01 compared with macrophages not treated with oxLDL in the same group, ^##^ *P* < 0.01 compared with the NC in the same group.

To test whether oxLDL-induced expression of DC-SIGN is crucial for the expression of inflammatory cytokines in macrophages, we compared the expression levels of IL-1β, MCP-1, TNF-α and MMP-9 after oxLDL treatment in macrophages transfected with DC-SIGN siRNA or negative control siRNA (NC). The knockdown efficiency of DC-SIGN is shown in Fig. 3A and B. DC-SIGN- or NC siRNA-transfected primary macrophages were incubated with 50 μg/m oxLDL for 6 hours. In the DC-SIGN knockdown and NC control macrophages, oxLDL treatment increased the mRNA expression of the cytokines measured. However, compared with macrophages transfected with NC, DC-SIGN-knockdown macrophages exhibited a much lower increase in the expression of IL-1β (DC-SIGN siRNA = 1.54-fold vs NC = 2.75-fold, *P* < 0.01), TNF-α (DC-SIGN siRNA = 1.76-fold vs NC = 3.67-fold, *P* < 0.01), MCP-1 (DC-SIGN siRNA = 1.64-fold vs NC = 3.41-fold, *P* < 0.01), and MMP-9 (DC-SIGN siRNA = 1.51-fold vs NC = 2.53-fold, *P* < 0.01) (Fig. 3C). The same trend was found in the ELISA results (Fig. 3D), suggesting that DC-SIGN participated in ox-LDL-mediated inflammatory cytokine expression.

### OxLDL treatment promotes the interaction of DC-SIGN with TLR4

**Figure 4:**
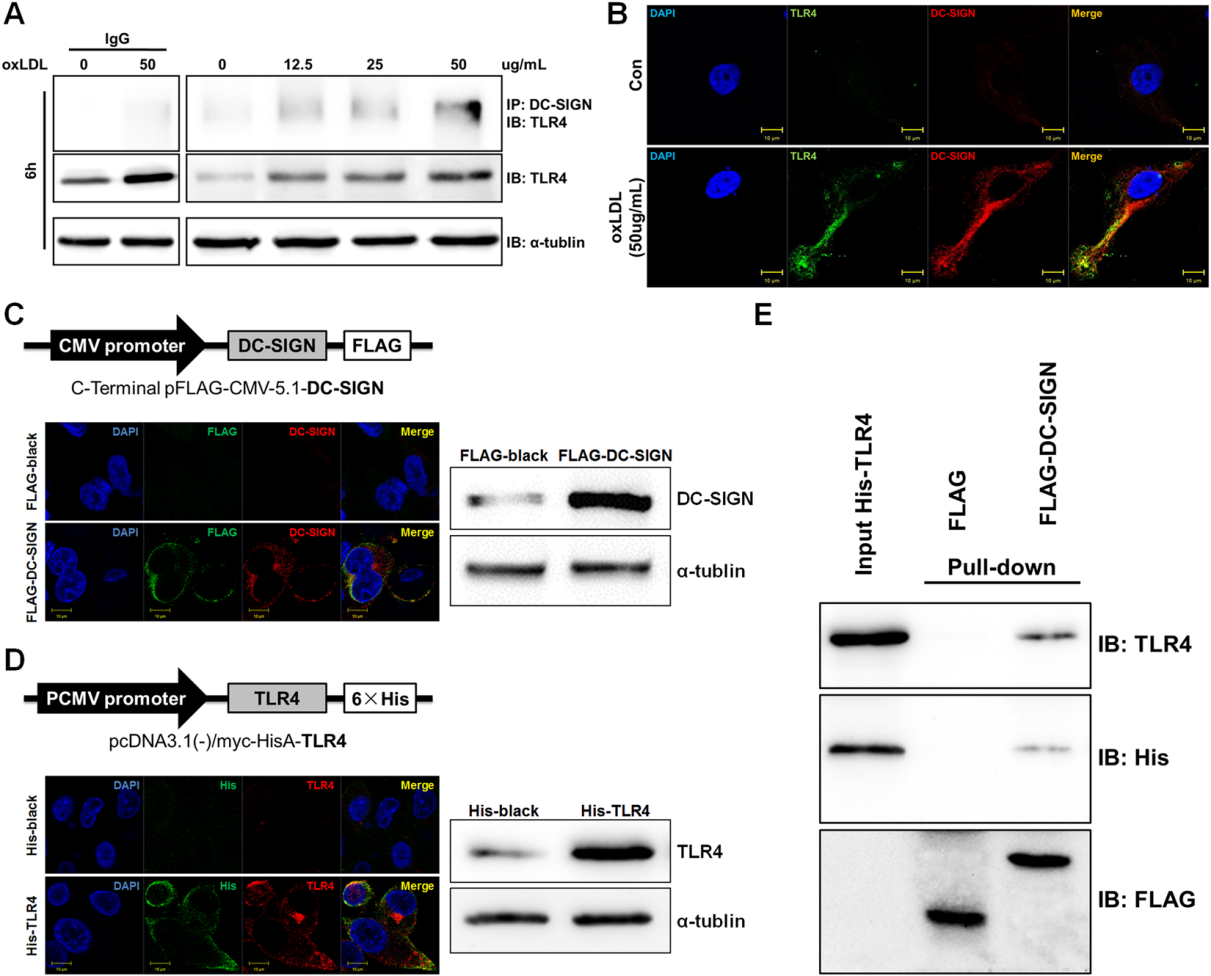
DC-SIGN interacted with and bound TLR4. (**A**) Macrophages were incubated with oxLDL (0, 12.5, 25 and 50 μg/ml) for 6 hours. Cell lysates were immunoprecipitated with a DC-SIGN antibody and probed with an antibody against TLR4. As loading controls, whole cell lysates were probed with antibodies against total TLR4 and α-tubulin. The IgG control had been detected in another Western-blot assay, where the protein level and exposure time were same Western-blot results on the right. (**B**) Macrophages were stimulated with or without 50 μg/ml oxLDL and stained with DC-SIGN (red) and TLR4 (green). Images were acquired by confocal microscopy (1,200x). Yellow indicates co-localization of the two proteins. (**C** and **D**) The maps of pFLAG-CMV-5.1-DC-SIGN and pcDNA3.1 (−)/myc-HisA-TLR4 are shown. pFLAG-CMV-5.1-DC-SIGN or pcDNA3.1 (−)/myc-HisA-TLR4 were transfected into HEK293 cells. The localization patterns of FLAG-DC-SIGN (FLAG: green, DC-SIGN: red) and His-TLR4 (His: green, TLR4: red) were detected by immunofluorescence stains, and the overexpression efficiency of FLAG-DC-SIGN and His-TLR4 were measured by western blot analysis. (**E**) *In vitro* pull-down of FLAG-DC-SIGN and His-TLR4 fusion proteins. The total cell lysate (10 μg) of FLAG and FLAG-DC-SIGN were absorbed onto anti-FLAG M2 beads and incubated with the whole cell lysate (10 μg) His-TLR4. Elutes were analyzed by SDS-PAGE followed by immunoblotting with anti-TLR4, anti-His and anti-FLAG. IB: Immunoblotting, IP: Immunoprecipitation.

The results of the immunofluorescence analysis in atherosclerotic lesions demonstrated that DC-SIGN co-localized with TLR4 in the CD68-positive regions (Fig. 1A). To verify whether oxLDL induced the endogenous interaction of DC-SIGN with TLR4 in macrophages, immunoprecipitation and immunofluorescence assays were performed. Immunoprecipitation assays showed that DC-SIGN interacted with TLR4 after oxLDL stimulation for 6 hours at different doses (12.5, 25 and 50 μg/ml) (Fig. 4A). Furthermore, some co-occurrence of DC-SIGN and TLR4 was observed upon oxLDL exposure (Fig. 4B). These results show that oxLDL-induced DC-SIGN formed a complex with TLR4.

Taken together, our results demonstrate that DC-SIGN co-expresses with TLR4 in the macrophages of plaque tissues and that oxLDL-induced DC-SIGN interacts with TLR4 *in vivo* and *in vitro*. Based on this conclusion, we tested whether the interaction between DC-SIGN and TLR4 shown by the immunoprecipitation assay could also be observed *in vitro* in a bimolecular interaction assay. The recombined plasmids of DC-SIGN and TLR4 were built to overexpress FLAG-DC-SIGN and His-TLR4 (Fig. 4C and D). The location and efficiency of FLAG-DC-SIGN and His-TLR4 overexpression was confirmed by immunofluorescence and western blot analysis (Fig. 4C and D). The recombined protein FLAG-DC-SIGN and FLAG were purified with anti-FLAG M2 beads *in vitro*. Then, the purified recombined protein FLAG-DC-SIGN and FLAG were incubated with His-TLR4 in an equilibration buffer. The immobilized FLAG-DC-SIGN fusion proteins (but not the immobilized FLAG) efficiently pulled down His-TLR4, as revealed by immunoblotting with anti-DC-SIGN, anti-His and anti-FLAG antibodies (Fig. 4E). These results show that DC-SIGN directly interacted with TLR4.

### DC-SIGN mediates TLR4-induced p65 activation

**Figure 5:**
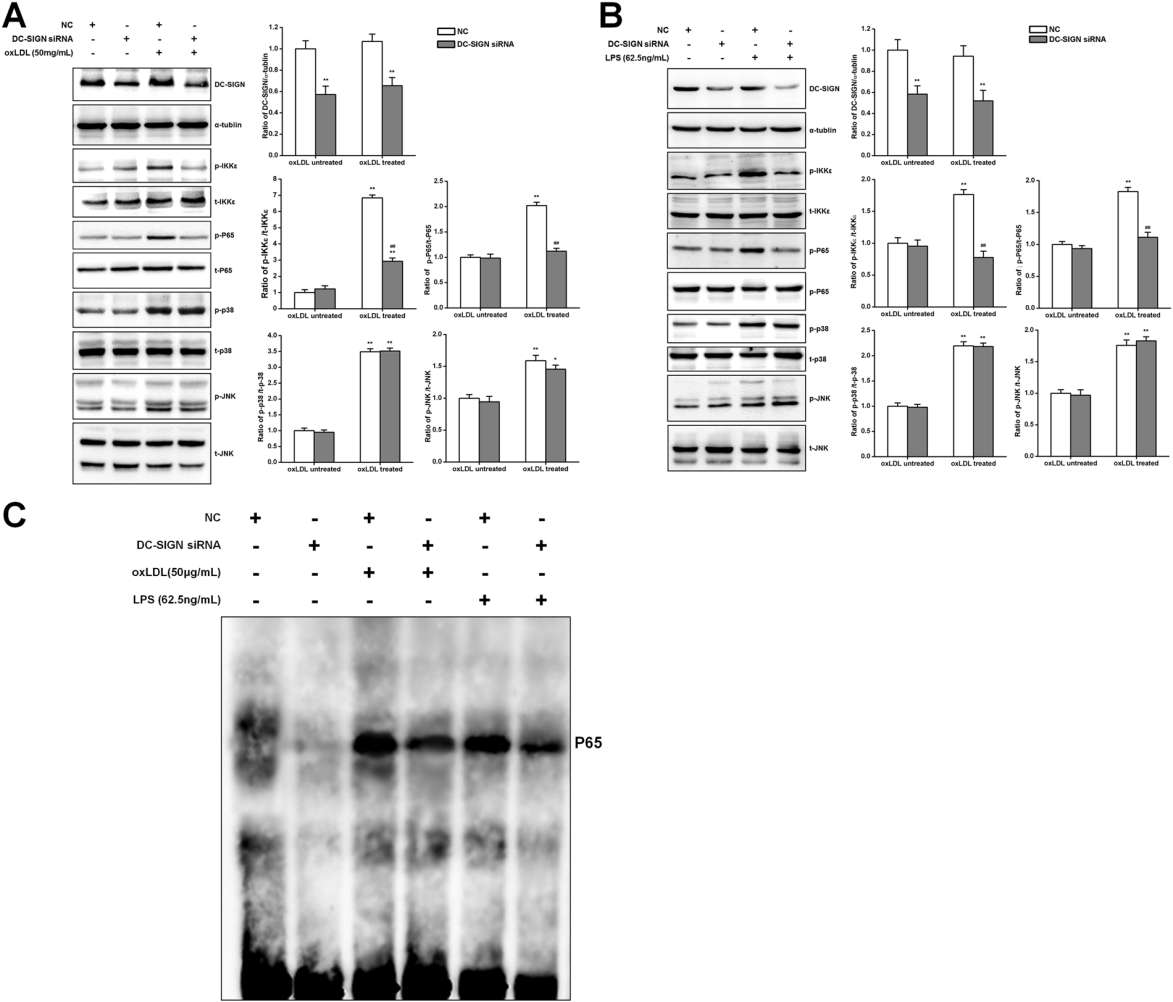
DC-SIGN participated in the TLR4-NFκB pathway. Negative control (NC) or DC-SIGN siRNA was transfected into macrophages treated or not treated with oxLDL (50 μg/ml) or LPS (62.5 ng/ml) for 60 min. Western blot analysis detected the knockdown efficiency of DC-SIGN and the phosphorylation of p38, JNK, IKKε and NFκB (**A** and **B**), which was quantified by densitometry in 3 independent experiments and presented as relative units (DC-SIGN/α-tubulin, p38, JNK, IKKε and NFκB phosphorylated protein/total protein). The data are expressed as the mean ± SD from 3 independent tests. *P < 0.05, **P < 0.01 compared with the macrophages not treated with oxLDL or LPS, ^##^ *P* < 0.01 compared with the NC in the same group. (**C**) Negative control (NC) or DC-SIGN siRNA was transfected into macrophages treated or not treated with oxLDL (50 μg/ml) or LPS (62.5 ng/ml) for 60 min. Nuclear extracts were then prepared and assayed for p65 activation by EMSA.

Base on the results of the *in vitro* pull-down assay, DC-SIGN is involved in the TLR4 signaling pathway. However, the p38, JNK, IKKε and NFκB pathways are known to be involved in TLR4 signaling, and all of these pathways regulate the inflammatory response in macrophages. To identify whether DC-SIGN regulates the TLR4-induced inflammatory response pathway, negative control (NC) and DC-SIGN siRNAs were transfected into macrophages (the top panel of Fig. 5A and B showing the efficiency of siRNA). After oxLDL and LPS stimulation for 60 min, the phosphorylation levels of p65, p38, IKKε and JNK were significantly increased (*P* < 0.05). Compared with the activation in the NC group, DC-SIGN knockdown significantly weakened the activation of p65 (oxLDL treatment: DC-SIGN siRNA = 1.12 ± 0.06 vs NC = 2.02 ± 0.07, *P* < 0.01; LPS treatment: DC-SIGN siRNA = 1.11 ± 0.08 vs NC = 1.83 ± 0.07, *P* < 0.01; Fig. 5A and B) and IKKε (oxLDL treatment: DC-SIGN siRNA = 2.93 ± 0.20 vs NC = 6.84 ± 0.18, *P* < 0.01; LPS treatment: DC-SIGN siRNA = 0.78 ± 0.10 vs NC = 1.76 ± 0.08, *P* < 0.01; Fig. 5A and B), but there was no effect on p38 and JNK activation (Fig. 5A and B). The EMSA results showed that knockdown of DC-SIGN inhibited LPS- and oxLDL induced-p65 activation (Fig. 5C). These results demonstrate that DC-SIGN mediated oxLDL- and LPS-induced TLR4 activation of p65 in macrophages.

## Discussion

In this study, we illustrated that DC-SIGN plays an important role in the macrophage inflammatory response. The *in vivo* study exhibited a stronger expression of DC-SIGN in the plaques of atherosclerotic patients than that in healthy controls. Soilleux *et al*.^16^ also found that DC-SIGN expression was increased, and DG-SIGN co-expressed with macrophage/DC lineage markers CD14, CD68, HLA-DR and S100 in plaques. Increased DC-SIGN is associated with the process of atherosclerosis. OxLDL is as key factor leading to the acute and chronic inflammatory response in atherosclerosis^30^, and it induced IL-1β, MCP-1, TNF-α and MMP-9 expression in macrophages. Our study showed that the highest dose and shortest stimulation time tested for oxLDL caused a rapid expression of DC-SIGN and inflammatory cytokines, which simulates the pathophysiological conditions of atherogenesis in which macrophages take up oxLDL and transform into foam cells to release inflammatory cytokines. In contrast, knockdown of DC-SIGN significantly reduced IL-1β, MCP-1, TNF-α and MMP-9 expression after oxLDL exposure and abolished oxLDL-induced NFκB activity. Our results illustrate that DC-SIGN plays a crucial role in the oxLDL-induced acute inflammation response of macrophages in atherosclerosis. However, we also mentioned that knockdown of DC-SIGN was not able to fully inhibit cytokine expression and secretion. OxLDL is known to regulate the activation of multiple receptors, such as TLR2, TLR4 and CD36, and all of these receptors and downstream signaling pathways participate in the inflammatory response^31^. Although DC-SIGN, as a pattern recognition receptor, is involved in the oxLDL induced-inflammatory response, it does not completely replace the function of other receptors.

Another interesting finding of our study is that DC-SIGN combines with TLR4 in macrophages to regulate the inflammatory response. In Fig. 1, DC-SIGN and TLR4 are shown to be co-localized at the region near the lipid core, which was enriched with macrophages (CD68-positive cells). Incubation with oxLDL promoted the endogenous interaction of DC-SIGN and TLR4 and their membrane expression in macrophages. This was further clarified by our *in vitro* pull-down assay showing that DC-SIGN directly binds to TLR4. In previous studies, it was found that DC-SIGN participated in the inflammatory response of TLR4 activation by exogenous infection^28^. Another study found that SIGN-R1 associates with TLR4 to capture LPS and induce signaling pathway activation to evoke innate macrophage responses^32^. *In vivo*, SIGN-R1-knockout mice have been shown to have a significantly reduced susceptibility to LPS-induced shock, and SIGN-R1/TLR4-knockout mice displayed a reduced susceptibility to experimental colitis relative to the severity of the disease observed in wild-type or TLR4-knockout mice. These data indicate that DC-SIGN is a critical innate factor in the response to LPS^33^.

The results of the oxLDL-induced DC-SIGN interaction with TLR4 were somewhat similar to those from LPS treatment. However, oxLDL is an atherosclerotic factor and not one associated with infection. The effects of oxLDL on DC-SIGN and TLR4 have not been previously reported and showed some differences from those of LPS. First, we found that oxLDL increased DC-SIGN expression in 6 hours and promoted the maximum amount of DC-SIGN/TLR4 complex formation in the same period. This indicates that DC-SIGN, after its rapid increase in expression, complexes with TLR4 and participates in the acute phase of the inflammatory response. Second, knockdown of DC-SIGN suppressed the oxLDL-enhanced phosphorylation of p65. This result demonstrates that DC-SIGN participates in the oxLDL-induced inflammatory response via the TLR4-NFκB axis. Third, the TLR4 signaling cascade in response to LPS is dependent on recruited adaptor proteins and can be broadly divided into myeloid differentiation factor 88 (MyD88)-dependent and MyD88-independent pathways, both of which lead to the activation of the NF-kB pathway and the expression of target pro-inflammatory genes^34–36^. P38 and JNK, as downstream components of the MyD88-dependent pathway, are involved in the NF-kB pathway. IKKε is a crucial molecule that is involved in the MyD88-independent pathway and regulates the activation of p65^37, 38^. In this study, we found that DC-SIGN knockdown only inhibited p65 and IKKε phosphorylation and not p38 and JNK phosphorylation. All of these results demonstrate that oxLDL regulates DC-SIGN binding to TLR4 to participate in the release of inflammatory cytokines via the NF-kB pathway but not the MyD88-dependent pathways.

In the LPS-induced TLR4 signaling pathway, LPS is extracted from bacterial membranes and released from vesicles by LPS binding protein (LBP). LBP then transfers LPS to CD14^39^, and CD14 splits the LPS aggregate into monomeric molecules and presents them to the TLR4–MD-2 complex, which leads to the activation of multiple signaling components, including NFκB and IRF3^40–42^. Thus, our data indicate that oxLDL promotes the binding of DC-SIGN to TLR4 to trigger the inflammatory response process. All of these results suggest that DC-SIGN has a similar function to CD14. It is likely that mechanisms other than those elucidated in this study also regulate the binding of DC-SIGN to TLR4, which will be clarified in our future studies.

In summary, we demonstrate the expression of DC-SIGN in macrophages of human atherosclerotic plaques and its co-localization with TLR4 in this study. Endogenous interactions and *in vitro* pull-down assays show that DC-SIGN directly binds to TLR4. DC-SIGN is involved in the oxLDL-induced TLR4 regulation of NF-kB activation and inflammatory cytokine expression. The implication of this study is that the deactivation of DC-SIGN or the dissociation of the DC-SIGN/TLR4 complex by synthetic chemicals may effectively attenuate the pathogenesis of atherosclerosis.

## Electronic supplementary material

Supplementary data
